# Gender disparities in prevalence by diagnostic criteria, treatment and mortality of newly diagnosed acute myocardial infarction in Korean adults

**DOI:** 10.1038/s41598-023-31014-y

**Published:** 2023-03-13

**Authors:** So Ree Kim, SungA Bae, Ji Yoon Lee, Min Sun Kim, Mi-Na Kim, Wook-Jin Chung, Jang-Ho Bae, Juneyoung Lee, Seong-Mi Park

**Affiliations:** 1 Division of Cardiology, Anam Hospital, Korea University College of Medicine, Seoul, Republic of Korea; 2 Division of Cardiology, Yonsei University College of Medicine, Yongin Severance Hospital, Yongin, Republic of Korea; 3 Department of Biostatistics, Korea University College of Medicine, Seoul, Republic of Korea; 4 Gachon Cardiovascular Research Institute, Gachon University, Incheon, Republic of Korea; 5 Division of Cardiology, Department of Internal Medicine, Konyang University Hospital, Daejeon, Republic of Korea; 6 Korea University Anam Hospital, Goryeodae-ro 73, Seongbuk-gu, Seoul, 02841 Republic of Korea

**Keywords:** Cardiology, Health care

## Abstract

Acute myocardial infarction (AMI) is highly prevalent and remains the leading cause of mortality. Particularly in women, under-recognition and management of AMI have been raised. The aim of this study was to investigate the long-term trends of prevalence, treatment methodologies, and mortality of AMI by gender. The subjects of this study were patients hospitalized for AMI according to the Korean National Health Insurance Claims Database from 2002 to 2018. Total 633,097 AMI patients were hospitalized, 40% women. The incidence of AMI has been increasing since 2011, with a lower incidence in women. Overall, 53.1% of patients underwent CAG, with a lower tendency in women than in men (39.8% vs. 62.3%). Furthermore, fewer women underwent PCI than men (77.5% vs. 85.8% in 2018, *p* < 0.0001). Of the 336,463 AMI patients undergoing CAG, women were undertreated with a lower prescription rate of beta-blockers or statins at discharge. When adjusted for age, women showed higher 7-day mortality but lower 1-year mortality relative to men. According to the Korean National Health Insurance Claims Database, women with AMI have been under-recognized, underdiagnosed, and undertreated in terms of revascularization or medical therapy for years suggesting that efforts to close the gender gap are necessary.

## Introduction

Medical advancements have significantly improved clinical outcomes in patients with acute myocardial infarction (AMI). Since Grüntzig performed the first percutaneous coronary angioplasty in 1977^1^, percutaneous coronary interventions (PCIs) have been performed worldwide and have improved the survival of patients with AMI^2,3^. Furthermore, the development of stents^4^ and adjunctive medical therapy such as potent antiplatelet agents^5,6^ have further improved clinical outcomes of AMI. However, AMI remains highly prevalent and is still the leading cause of mortality^7,8^.

Several reports highlight the fact that although major progress has been made in reducing cardiovascular disease mortality in women, medical research has historically neglected the health needs of women. For example, women with AMI face under-recognition and under-management^9^, with a lower tendency to be treated with guideline-directed medical therapies^10–12^, cardiac catheterization^10–13^, and timely reperfusion^14^. Although newer studies have suggested improvements in these sex-related disparities^15^, real-world data regarding the patterns of diagnosis, treatment, and prognosis of AMI by gender are limited. The purpose of our study was to investigate the long-term trends of AMI prevalence by diagnostic criteria, treatment methodologies, and mortality of AMI by gender.

## Methods

### Data source and data extraction

The data used for this study were obtained from the Korean National Health Insurance Service (KNHIS). The KNHIS, as the single insurer of the Korean National Health Insurance Program (KNHIP), is currently operating a medical claim database. All medical service providers and the Korean population are obligated to join the KNHIP according to national acts. Therefore, the KNHIS database covers almost all medical procedures performed in the entire Korean population since 2002. The database is based on International Statistical Classification of Diseases and Related Health Problems (ICD) 10 code system.

The KNHIS database contains principal and secondary diagnoses, hospitalization/outpatient treatment, medical fees, contents of medical services, prescribed medications, patients’ sexes, and ages, date of death, among other information, which are arrayed according to the claims made by medical institutions. We arranged the medical records based on individual patients and extracted the years of hospitalization from the data of the hospitalized AMI cases for analysis. We used the structured identification code provided by the KNHIS to identify individual patients. The KNHIS erased all private information, such as name, social security number, address, and phone number, from the original patient claim data. The study protocol was approved and a waiver of informed consent was obtained by the Institutional Review Board of Korea University Anam Hospital [Seoul, Korea] (IRB number: 2020AN0148). All methods were carried out in accordance with relevant guidelines and regulations.

### Definition of AMI

The subjects of this study were patients who were hospitalized for AMI (Code “I21” of the ICD-10) from January 1, 2003, to December 31, 2018. Although the KNHIS data contains principal and secondary diagnoses, we chose a 1-year washout period to extract true incident cases (2002). We selected patients hospitalized for principal diagnosis each year. Given the characteristics of AMI, we excluded those who received only outpatient treatment, but not hospital treatment as they were not expected to be newly diagnosed. As there were cases in which some hospitalized patients were readmitted due to relapses or complications, we regarded such patients as having disease onset in the year of their first hospitalization and excluded them if they were subsequently hospitalized. We further excluded patients who did not undergo cardiac enzyme testing (troponin T, I or creatine kinase-MB—C3941, C3942, CY277, CY278, CY279, D4021, D4022, D4023, B2640, D4040, D4061, and D4062). In addition, patients who were diagnosed with AMI by coronary angiography (CAG—HA670, HA680, HA681, and HA682) were identified for further analysis.

### Study variables

Demographic variables including age, sex, comorbidities, smoking status, and type of AMI (ST-elevation myocardial infarction [MI] (I21.3), non-ST-elevation MI (I21.4), and unspecified) were assessed. Comorbidities included hypertension, diabetes, dyslipidemia, atrial fibrillation, cerebral infarction, cerebral hemorrhage, coronary artery disease, and any cancer. The presence of the above disease codes within 1 year before or after the diagnosis of AMI was considered a comorbidity. Smoking status was assessed using data from the national health screening performed within 3 years before the diagnosis of AMI.

The performance rates of CAG and PCIs were assessed. Severe clinical conditions included intubated status, extracorporeal membrane oxygenation, intra-aortic balloon pump, or continuous renal replacement therapy. Medications including beta-blockers and high-intensity statins, which were prescribed for more than 30 days within one month of AMI, were assessed at discharge. Definitions by code are presented in Supplementary Table 1.

The primary outcome variables were the in-hospital and 1-year mortality rates, defined as the proportion of patients with AMI who died during admission and within 1 year of follow-up after admission, respectively, against the number of cases diagnosed with the disease. The 3-day, 7-day, and 30-day mortality rates were also analyzed.

### Statistical analysis

Data are presented as frequencies (percentages) for categorical variables or means ± standard deviations for continuous variables. The chi-square test or Student’s t-test was used to compare the baseline characteristics between men and women, and the incidence of mortality per 100 person-years was estimated. To estimate the 3-day, 7-day, 30-day, and 1-year mortality for, both, men and women, Kaplan–Meier analysis was performed, and a log-rank test was used to compare overall mortality. A multivariable Cox proportional hazards regression model was used to compare the clinical outcomes between men and women after adjusting for age, which showed a significant difference. Thus, the proportional hazard assumption for this model between men and women was satisfied. The incidence of AMI was estimated using the national population for aged more than or equal to 20 years, which was obtained from the Korean Statistical Information Service as denominator. The trend of incidence across years was examined using the Cochran–Armitage test. All data analyses were conducted using SAS version 9.4 (SAS Institute, Cary, NC, USA), and a two-sided *p* value < 0.05 was considered to be statistically significant.

## Results

### Incidence rate of AMI

**Figure 1:**
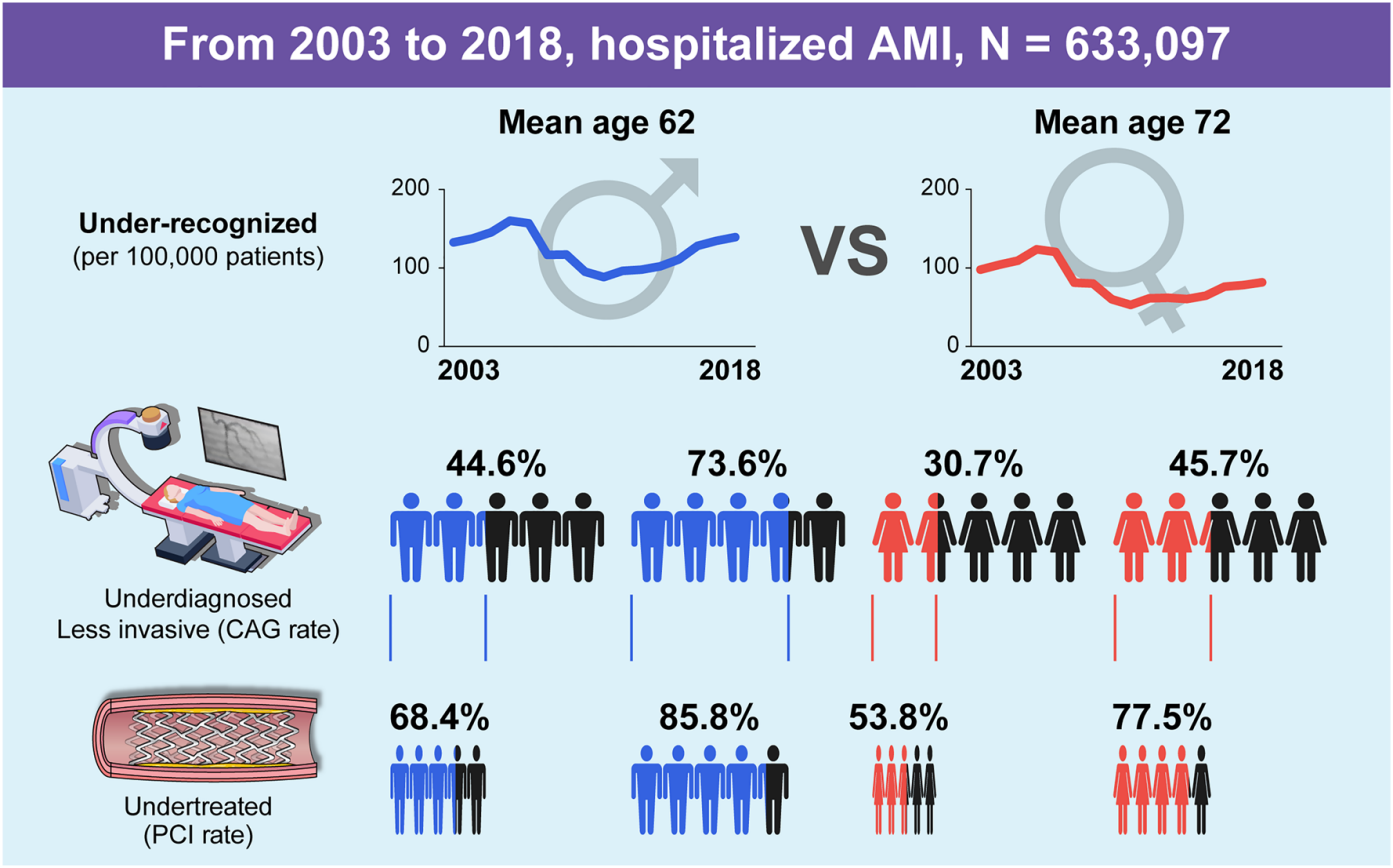
Incidence, CAG, and PCI rate of patients with AMI by gender from 2003 to 2018 according to the Korean National Health Insurance Claims Database. The CAG rate was defined as the proportion of patients diagnosed with AMI who also underwent CAG compared to the number of patients diagnosed with the disease. The PCI rate was defined as the proportion of patients diagnosed with AMI who underwent PCI against the number of patients diagnosed with the disease and undergoing CAG. Abbreviations: AMI, acute myocardial infarction; CAG, coronary angiography; PCI, percutaneous coronary intervention.

From 2003 to 2018, 633,097 patients were hospitalized for AMI. Approximately 40% of patients were women. The incidence of hospitalized AMI patients increased between 2011 and 2018, from 71 per 100,000 to 110 per 100,000 patients, and women were less likely to be hospitalized for AMI (53 vs. 88 in 2011, 82 vs. 139 per 100,000 patients in 2018, *p* ≤ 0.0001), with gender-disparities for hospitalization increasing (Fig. 1).

### AMI undergoing CAG

**Figure 2:**
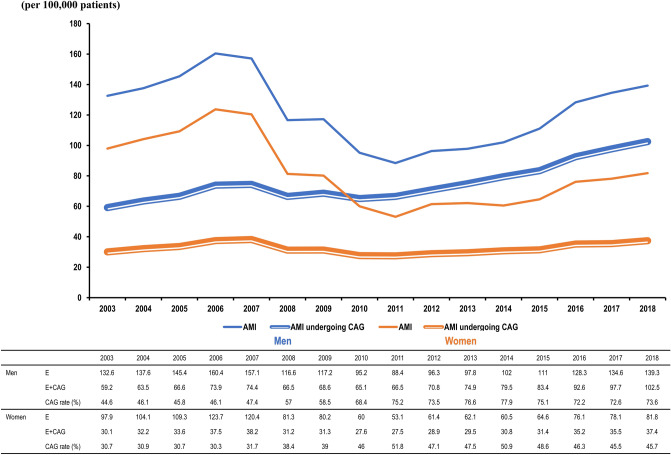
Incidence of AMI and CAG rate from 2003 to 2018 according to the Korean National Health Insurance Claims Database. The CAG rate was defined as the proportion of patients diagnosed with AMI who also underwent CAG compared to the number of patients diagnosed with the disease. Since 2003, fewer women with AMI have undergone CAG than men, with a more prominent gender gap. Abbreviations: AMI, acute myocardial infarction; CAG, coronary angiography; E, cardiac enzyme.

Overall, 53.1% of the patients underwent CAG. Women were less likely to undergo CAG; only 39.8% of women compared to 62.3% of men underwent CAG (Supplementary Fig. 1). Since 2003, fewer women with AMI have undergone CAG than men. In male patients with AMI, the CAG rate consistently increased from 44.6% in 2003 to 68.4% in 2010, reaching 73.6% in 2018, whereas that of women with AMI remained relatively unchanged from 30.7% in 2003, 46.0% in 2010, and 45.7% in 2018. The gender disparity between patients undergoing invasive procedures has continued to increase since 2010 (Figs. 1, 2).

### Baseline characteristics

Table 1 shows the baseline characteristics of the 336,463 patients with AMI who underwent CAG. ST-elevation MI and non-ST-elevation MI comprised 26% and 19% of the overall AMI patients, respectively, and the others were categorized as unspecified MI. Seventy percent of the patients were male, and two-thirds of the patients were older than 60 years, with women being much older than men (mean age: 65; 71.5 ± 11.2 vs. 62.0 ± 12.3, *p* < 0.0001). Hypertension and dyslipidemia were present in approximately 80% of the patients, while diabetes was present in 54% of the patients. Women were more likely to be hypertensive or diabetic and had a history of cerebrovascular infarction. Men were more likely to be current smokers and have coronary artery disease.

**Table 1:** Baseline characteristics.

	Total	Men	Women	*p* value
	(N = 336,463)	(N = 234,281)	(N = 102,182)	
Age	64.9 ± 12.7	62.0 ± 12.3	71.5 ± 11.2	< .0001
< 60	118,067 (34.97)	101,768 (43.44)	15,099 (14.78)	
≥ 60	219,596 (65.03)	132,513 (56.56)	87,083 (85.22)	
AMI type				< .0001
STEMI	88,445 (26.19)	65,832 (28.10)	22,327 (21.85)	
NSTEMI	65,596 (19.43)	44,927 (19.18)	20,516 (20.08)	
Unspecified	183,622 (54.38)	123,522 (52.72)	59,339 (58.07)	
Hypertension	272,653 (80.75)	182,652 (77.96)	88,991 (87.09)	< .0001
Diabetes	180,896 (53.57)	119,897 (51.18)	60,343 (59.05)	< .0001
Dyslipidemia	267,638 (79.26)	186,219 (79.49)	80,513 (78.79)	< .0001
Atrial fibrillation	26,892 (7.96)	16,893 (7.21)	9881 (9.67)	< .0001
Cerebrovascular infarction	45,498 (13.47)	27,357 (11.68)	17,972 (17.59)	< .0001
Cerebrovascular hemorrhage	2415 (0.72)	1548 (0.66)	858 (0.84)	< .0001
Coronary artery disease	60,417 (17.89)	43,052 (18.38)	17,140 (16.77)	< .0001
Cancer	23,469 (6.95)	16,540 (7.06)	6849 (6.70)	< .0001
Current smoker	60,747 (17.99)	57,913 (24.72)	2729 (2.67)	< .0001

AMI, acute myocardial infarction; NSTEMI, non-ST-elevation myocardial infarction; STEMI, ST-elevation myocardial infarction.

### AMI undergoing PCI

**Figure 3:**
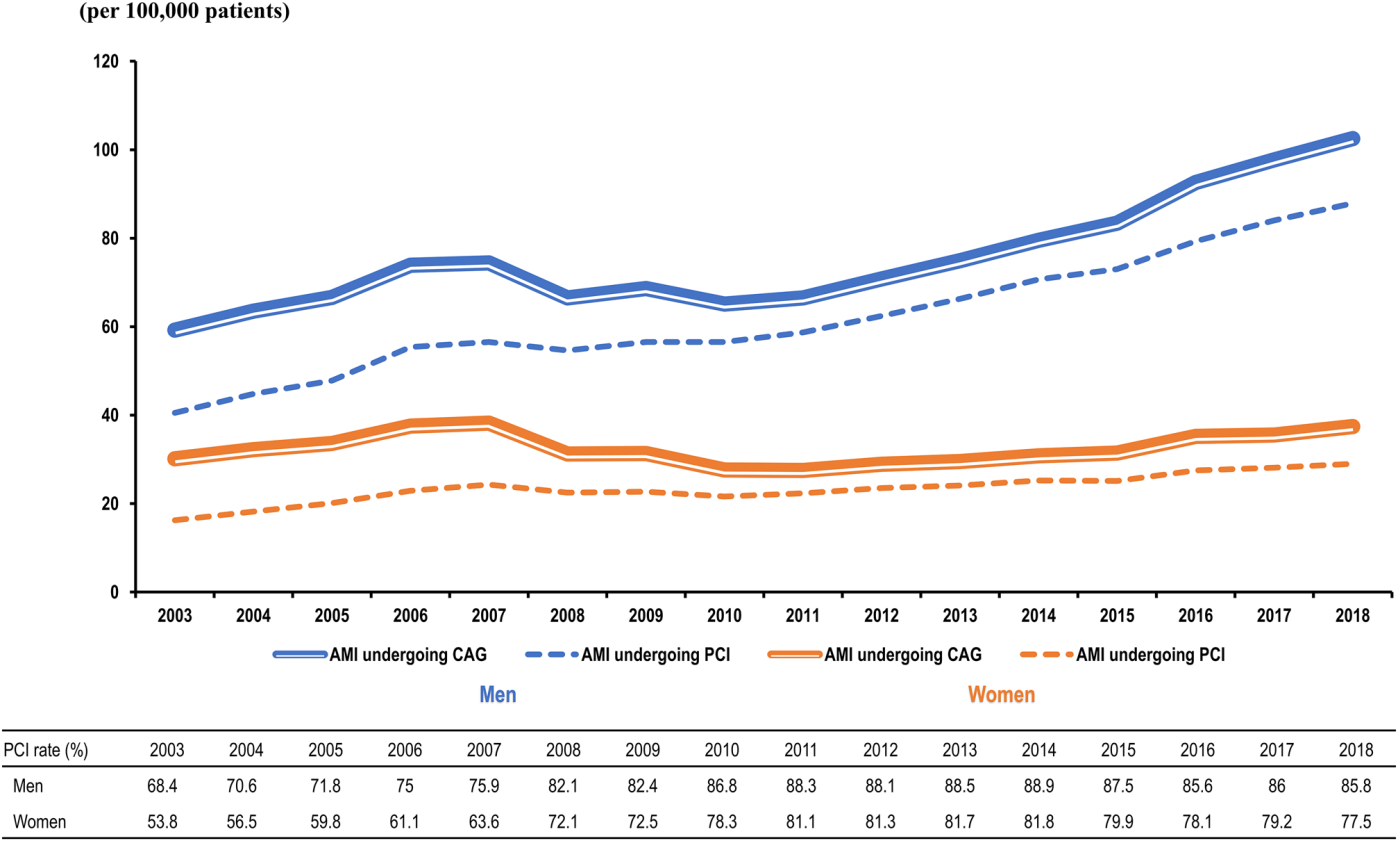
Incidence of AMI undergoing CAG and patients who underwent PCI from 2003 to 2018 according to the Korean National Health Insurance Claims Database. The PCI rate was defined as the proportion of patients diagnosed with AMI who underwent PCI against the number of patients diagnosed with the disease and undergoing CAG. Abbreviations: AMI, acute myocardial infarction; CAG, coronary angiography; PCI, percutaneous coronary intervention.

In total, more than 85% of AMI patients who were recommended CAG underwent PCI. However, women were less likely to undergo PCI than men (Figs. 1 and 3). Since 2011, the PCI rate has been significantly lower in women (81.1% vs. 88.3% in 2011, 77.5% vs. 85.8% in 2018, *p* ≤ 0.0001), and this gender gap in PCI rate has been larger.

### AMI with severe clinical condition

In patients with AMI, severe clinical conditions defined by intubated status or status on extracorporeal membrane oxygenation, intra-aortic balloon pump, or continuous renal replacement therapy accounted for 13.1% of total AMI cases in 2018. This rate has remained stationary since 2010 (Supplementary Fig. 2). Women with AMI were more likely to have severe clinical conditions (14.6% vs. 12.5% in 2018, *p* < 0.0001), and this difference has persisted for over 10 years (Supplementary Fig. 2). While AMI patients stayed in the hospital for a median of six days, patients with severe clinical conditions stayed in the hospital for 11 days in women and 12 days in men (*p* = 0.0589).

### Medication at discharge

Approximately 68% of the patients were taking beta-blockers and 85% were taking statins at discharge in 2018 (Supplementary Figs. 3 and 4). The prescription rate of beta-blockers has been decreasing from 78.3% since 2012 (*p* trend ≤ 0.0001), while that of statins has not changed since 2012 (*p* trend = 0.5231). Women were less likely to receive beta-blockers (62.6% vs. 69.7% in 2018, *p* < 0.0001) and statins (79.8% vs. 87.2% in 2018, *p* ≤ 0.0001) at discharge compared to men (Supplementary Figs. 3 and 4). This gender disparity in the prescription rate of beta-blockers and statins has persisted for over a decade.

### Clinical outcomes

**Figure 4:**
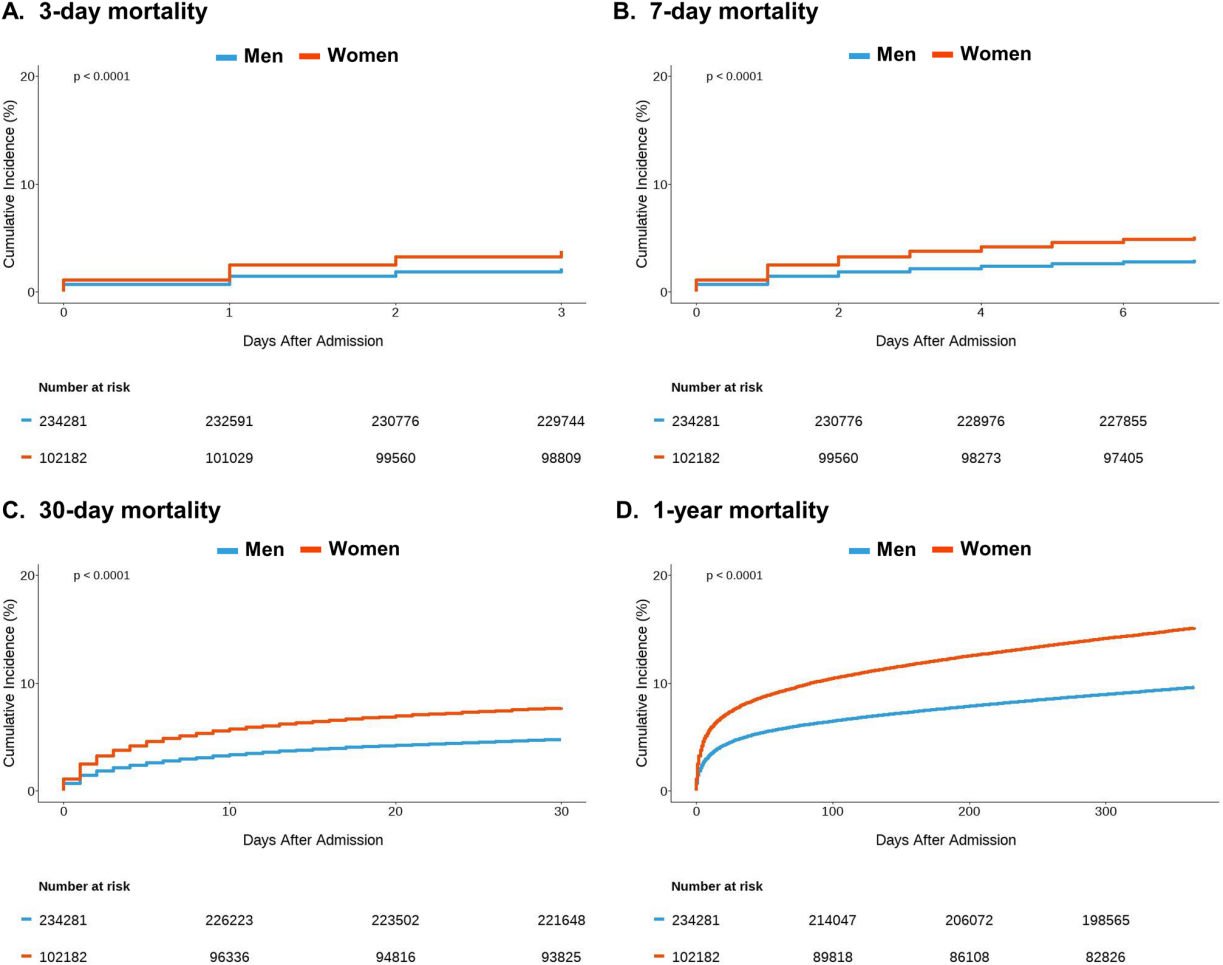
Gender differences in clinical outcomes. Kaplan–Meier curves of unadjusted 3-day (**A**), 7-day (**B**), 30-day (**C**), and 1-year (**D**) mortality.

In-hospital mortality has increased since 2003 from 2.2% to 5.3% (*p* trend < 0.0001). Thirty-day mortality and 1-year mortality also increased slightly since 2003 (*p* trend ≤ 0.0001), reaching 6.9% and 12.9% in 2018, respectively (Supplementary Fig. 5). Gender differences in clinical outcomes are shown in Fig. 4 and Supplementary Table 2. Women with AMI showed elevated short- and long-term mortality than men. Women demonstrated increased 3-day (470 vs. 270 per 100 person-years, unadjusted hazard ratio [HR] (95% confidence interval [CI]) 1.74 (1.67–1.81), *p* < 0.0001) and 7-day mortality (278 vs. 158 per 100 person-years, unadjusted HR (95% CI) 1.75 (1.69–1.82), *p* < 0.0001). When adjusted for age, women still showed a higher 3-day (adjusted HR (95% CI) 1.07 (1.02–1.12), *p* = 0.0038) and 7-day mortality (adjusted HR (95% CI) 1.08 (1.04–1.12), *p* = 0.0002) than men. However, there was no significant difference in 30-day mortality (adjusted HR (95% CI) 0.99 (0.96–1.02, *p* = 0.3478). Furthermore, 1-year mortality was especially lower in women (adjusted HR (95% CI) 0.91 (0.89–0.93, *p* < 0.0001) than in men.

## Discussion

This study demonstrates the long-term trends of the prevalence by diagnostic criteria, treatment, and mortality of AMI by gender according to the Korean National Health Insurance Claims Database from 2002 to 2018. While the incidence of AMI has been increasing since 2011, fewer women are hospitalized for AMI compared to men. Furthermore, women with AMI were less likely to undergo CAG and PCI than men, and this gender disparity for diagnosis and treatment has only become more prominent. Despite more severe clinical manifestations in women, women were under-treated with a lower prescription rate of beta-blockers or statins at discharge than men. In general, in-hospital, 30-day, and 1-year mortality rates of AMI have increased over the past decade, reaching 5.3%, 6.9%, and 12.9% in 2018, respectively. When adjusted for age, women showed higher short-term mortality rates but lower 1-year mortality rates than men.

AMI is the leading cause of ischemic heart disease, which is a great global mortality burden^16^. In the United States, the rate of AMI hospitalization was 1,485 per 100,000 person-years in 2002, but has since declined to 1,122 per 100,000 person-years in 2011^7^. Similarly, a significant decline in AMI event rates were observed in Europe between 1985 to 2010^17^. However, trends of the incidence of AMI were different in the present study, which has been increasing between 2011 and 2018. Moreover, despite the downward trends globally, AMI is still the leading cause of mortality worldwide^18^. In the present study, in-hospital, 30-day, and 1-year mortality from AMI has increased since 2011. Korea implemented a comprehensive plan for cardiovascular disease from 2006 to 2010 through the creation of regional cardiovascular centers throughout the country to reduce the average waiting time from emergency room arrival to catheterization initiation. As a result, more patients were diagnosed with AMI and underwent primary PCI; meanwhile, patients with more severe forms of AMI presented at the hospital, which may have resulted in the increased mortality of hospitalized AMI patients since 2011^19^.

In particular, women are at a higher risk of mortality due to AMI than men, with more atypical symptoms such as shortness of breath rather than chest pain^20^, lower chance of undergoing CAG or PCI^21,22^, longer door-to-balloon time, and inadequate cardiovascular medications^23^. In the present study, women with AMI were under-recognized and underdiagnosed, which is in line with the previous studies^21,22^. As women with AMI were older and had more comorbidities, medical treatment may have been preferred over invasive procedures. In addition, unusual mechanisms, including coronary microvascular spasm and distal microembolization, may have contributed to the pathophysiology of nonobstructive AMI in women^24^. However, as women have greater resistance to cardiomyocyte loss than men^25^, greater salvage with PCI should be considered in women after acute coronary ischemia^26,27^.

Medical therapy for AMI also differed between gender in this study. While high-intensity statin was essential for secondary prevention of AMI^28–31^, only 79.8% of women were prescribed statins with a difference of -7.4% from that of men in this study. In addition, in most trials, women were more likely to discontinue statins due to adverse events. Despite the beneficial effect on clinical outcomes^32,33^, beta-blockers were also under-prescribed in women, which was in line with a previous study^10^. Some explanations for this gender gap in medical therapy in AMI are as follows. Women may be less tolerant to medical therapies owing to their side effects. As women are more likely to have an unusual pathophysiology of AMI^24^ and non-selective beta-blockers should be avoided in coronary arterial vasospasm, this might preclude physicians from prescribing beta-blockers to women.

As severe forms of AMI are more prevalent in women, short-term outcomes are likewise much worse in women. Women were diagnosed with AMI at an older age and had more atypical symptoms than men. This might result in severe clinical presentations of AMI and a lower PCI rate, resulting in worse short-term outcomes in women. Interestingly, the age-adjusted 1-year outcomes were better in women than in men. In the Korea Acute Myocardial Infarction Registry-National Institute of Health registry including AMI patients following PCI, women showed higher unadjusted 1-year mortality than men (8.6% vs. 4.7%: HR (95% CI) 1.863 (1.578–2.199), *p* ≤ 0.001)^34^. However, after propensity score matching, women showed similar 1-year mortality (8.4% vs. 9.6%, HR (95% CI) 0.879 (0.681–1.135), *p* = 0.323) relative to men. This suggests that if the initial management of AMI is appropriate, women may have a comparable long-term prognosis. The reason for the poor long-term prognosis in men is not clear, but it is presumed to be related to social factors, such as continued smoking after recovering from AMI.

This study has several limitations. As this study was based on the Korean National Health Insurance Claims Database, it is possible that the diagnosis of AMI might not have been clear when the disease code was entered incorrectly. Compounding factors could not be adjusted for in the analysis because details of the demographic and angiographic data were not included. In terms of clinical outcomes, the major components of adverse cardiac and cerebrovascular events, including target lesion revascularization, nonfatal MI, and nonfatal stroke, were not available. Therefore, only mortality data were available for this study. However, as a large number of patients were included, gender differences in clinical outcomes could be observed.

In conclusion, according to the Korean National Health Insurance Claims Database, women with AMI are under-recognized, underdiagnosed, and undertreated in terms of revascularization or medical therapy. Gender disparities in the prevalence, treatment, and clinical outcomes of AMI have become more prominent in recent years. To close the gender gap in AMI, more attention is needed to reinforce education, administer nationwide campaigns, and implement systematic protocols.

## Supplementary Information

Supplementary Information.

## Data Availability

The data that support the findings of this study were provided by Korean National Health Insurance Claims Database. Restrictions apply to the availability of these data, which were used under license for the current study, and so are not publicly available. However, requests for the use of data may be made at Korean National Health Insurance Claims Database. If someone wants to request the data from this study, S.-M. Park should be contacted.

## Abbreviations

AMI: Acute myocardial infarction
CAG: Coronary angiography
ICD: International Statistical Classification of Diseases
KNHIS: Korean National Health Insurance Service
PCI: Percutaneous coronary intervention
