# Supplementary material for: Gender disparities in prevalence by diagnostic criteria, treatment and mortality of newly diagnosed acute myocardial infarction in Korean adults
Source: Sci Rep. 2023 Mar 13;13:4120. doi: 10.1038/s41598-023-31014-y (PMC10011387; doi:10.1038/s41598-023-31014-y)
Supplement: Supplementary file 1 — Supplementary Information. [file 41598_2023_31014_MOESM1_ESM.pdf]

Gender Disparities in Prevalence by Diagnostic Criteria, Treatment and Mortality of Newly Diagnosed Acute Myocardial Infarction in Korean Adults

So Ree Kim, MD[1†], SungA Bae, MD, PhD[2†], Ji Yoon Lee[3], Min Sun Kim[3], Mi-Na Kim, MD, PhD[1], Wook-Jin Chung, MD, PhD[4], Jang-Ho Bae, MD, PhD[5], Juneyoung Lee[3], Seong-Mi Park, MD, PhD[1*]

Supplementary Table 1. Definitions by Codes

Supplementary Table 2. Clinical Outcomes

Supplementary Figure 1. Study flow

Supplementary Figure 2. Incidence of AMI with Severe Clinical Condition from 2003 to 2018

Supplementary Figure 3. Prescription Rate of Beta Blockers at Discharge in Patients with AMI undergoing PCI

Supplementary Figure 4. Prescription Rate of Statins at Discharge in Patients with AMI undergoing PCI

Supplementary Figure 5. Clinical Outcomes of Patients with AMI from 2003 to 2018 according to the Korean National Health Insurance Claims Database

Supplementary Table 1. Definitions by Codes

	Codes
**Hypertension**	I10, I11, I12, I13, I14, I15
**Diabetes**	E10, E11, E12, E13, E14
**Dyslipidemia**	E78
**Atrial fibrillation**	I48
**Cerebral infarction**	I63, I69
**Cerebral hemorrhage**	I61
**Coronary artery disease**	I25.1
**Any cancer**	C~
**Percutaneous coronary intervention**	M6551, M6552, M6553, M6554, M6561, M6562, M6563, M6564, M6565, M6566, M6567, M6571, M6572, M6634, M6638
**Intubated status**	M5859, M5861, M5862, M5863, M5864, M5865, M5866, M5867, M5868, M5830, M5850, M5857, M5858, M5860
**Extracorporeal membrane oxygenation**	O1901, O1902, O1903, O1904, O1905, O1906
**Intra-aortic balloon pump**	O1921, O1922
**Continuous renal replacement therapy**	O7031, O7032, O7033, O7034, O7035, O7051, O7052, O7053, O7054
**Beta-blockers**	117901ATB, 117902ATB, 117903ATB, 117904ATB, 125001ATB, 125002ATB, 125003ATB, 125004ACR, 125005ATB, 125006ACR, 125007ACR, 125008ACR, 662201ATB, 662202ATB, 193802ATB, 193803ATB, 489501ATB, 489502ATB, 489503ATB, 683000ATB, 683100ATB, 683200ATB, 111402ATB, 111403ATB, 483101ATB, 483102ATB, 219901ATB, 219904ATB

**High-intensity statins**	111503ATB, 111504ATB, 454002ATB, 502203ATB, 502204ATB, 634600ATB, 634800ATB, 640900ATB

Supplementary Table 2. Clinical Outcomes

	Events (n)	Follow-up duration (year)	Incidence rate (per 100 person-years)	Unadjusted		Adjusted (age)	
				HR (95% CI)	p-value	HR (95% CI)	p-value
3-day mortality							
Men (N=234,281)	5,129	1897.63	270.28 (262.99, 277.78)	1 (reference)		1 (reference)	
Women (N=102,182)	3,855	819.71	470.29 (455.68, 485.37)	1.74 (1.67, 1.81)	<.0001	1.07 (1.02, 1.12)	0.0038
7-day mortality							
Men (N=234,281)	6,948	4396.26	158.04 (154.37, 161.8)	1 (reference)		1 (reference)	
Women (N=102,182)	5,251	1889.04	277.97 (270.55, 285.59)	1.75 (1.69, 1.82)	<.0001	1.08 (1.04, 1.12)	0.0002
30-day mortality							
Men (N=234,281)	11,226	18495.96	60.69 (59.58, 61.83)	1 (reference)		1 (reference)	
Women (N=102,182)	7,914	7875.40	100.49 (98.3, 102.73)	1.64 (1.6, 1.69)	<.0001	0.99 (0.96, 1.02)	0.3478
1-year mortality							
Men (N=234,281)	22,100	207922.53	10.63 (10.49, 10.77)	1 (reference)		1 (reference)	
Women (N=102,182)	15,214	87179.07	17.45 (17.18, 17.73)	1.62 (1.59, 1.66)	<.0001	0.91 (0.89, 0.93)	<.0001

Abbreviations: HR, hazard ratio; CI, confidence interval

Supplementary Figure 1. Study flow

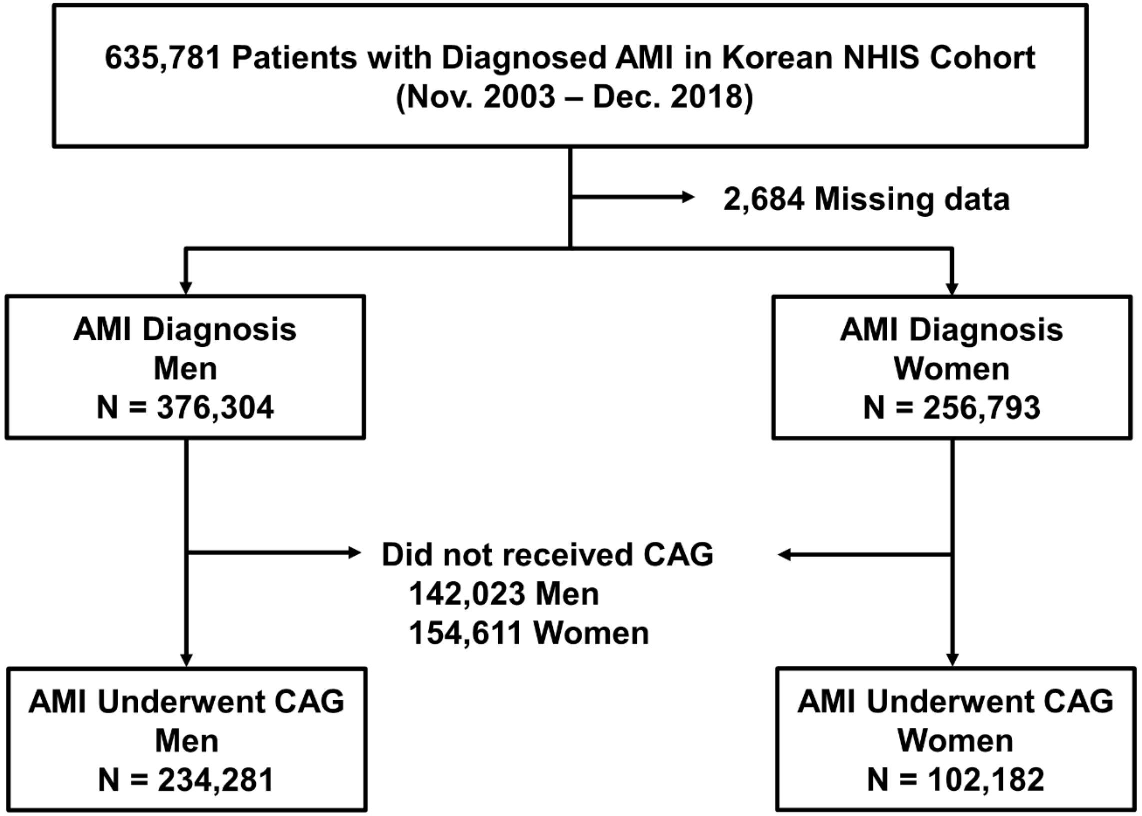

From November 2003 to December 2018, 635,781 patients were diagnosed with AMI by International Statistical Classification of Diseases and Related Health Problems 10 code system. After excluding 2,684 patients with missing data, 633,097 hospitalized patients with AMI were analyzed based on data from the Korean National Health Insurance Service. Among 376,304 men and 256,793 women, 142,023 (37.7%) and 154,611 (60.2%) did not undergo CAG, respectively. Accordingly, 336,463 patients with AMI who underwent CAG were further analyzed.

Supplementary Figure 2. Incidence of AMI with Severe Clinical Condition from 2003 to 2018

A. Incidence of AMI with Severe Clinical Condition

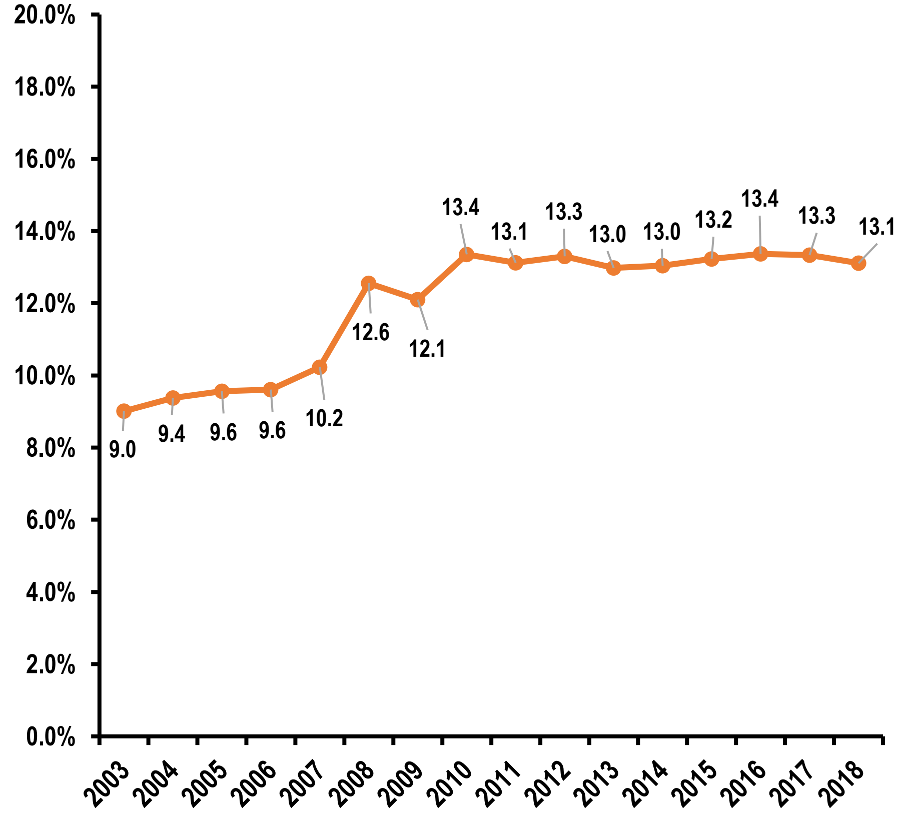

B. Gender difference

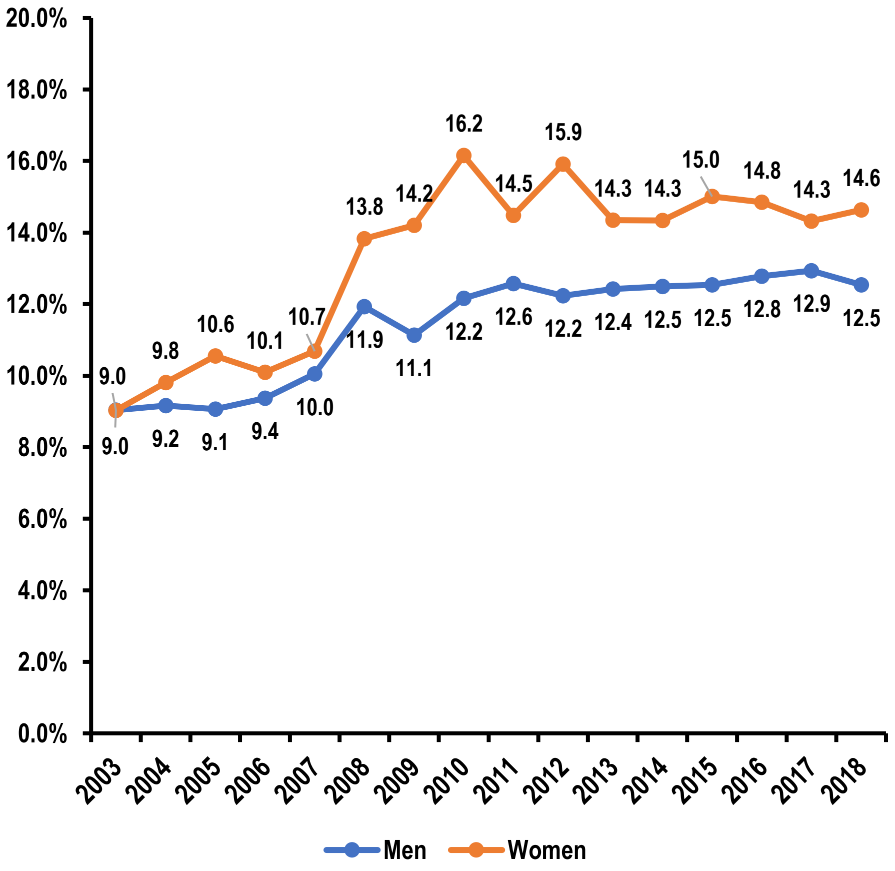

Supplementary Figure 3. Prescription Rate of Beta Blockers at Discharge in Patients with AMI undergoing PCI

A. Total patients with AMI

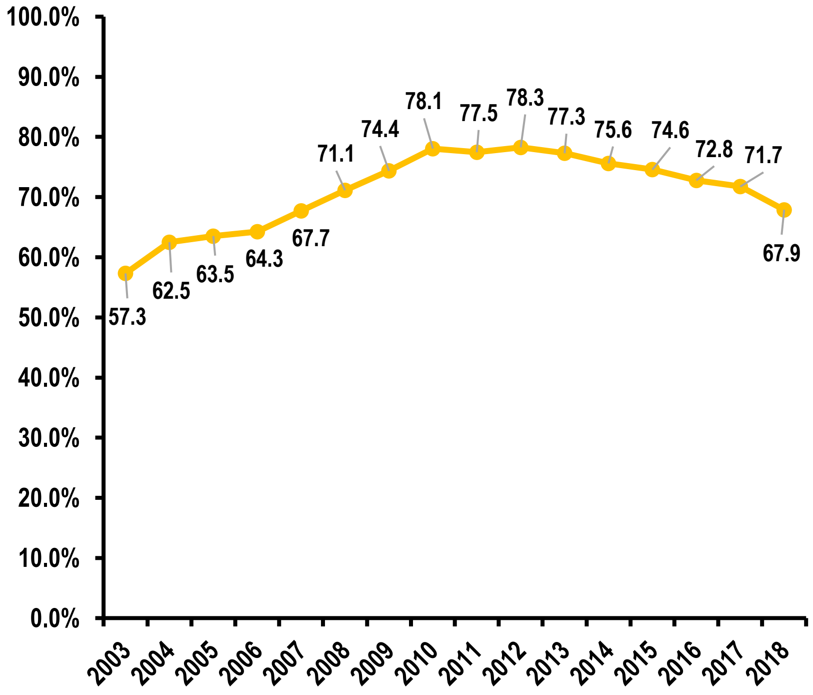

B. Gender difference

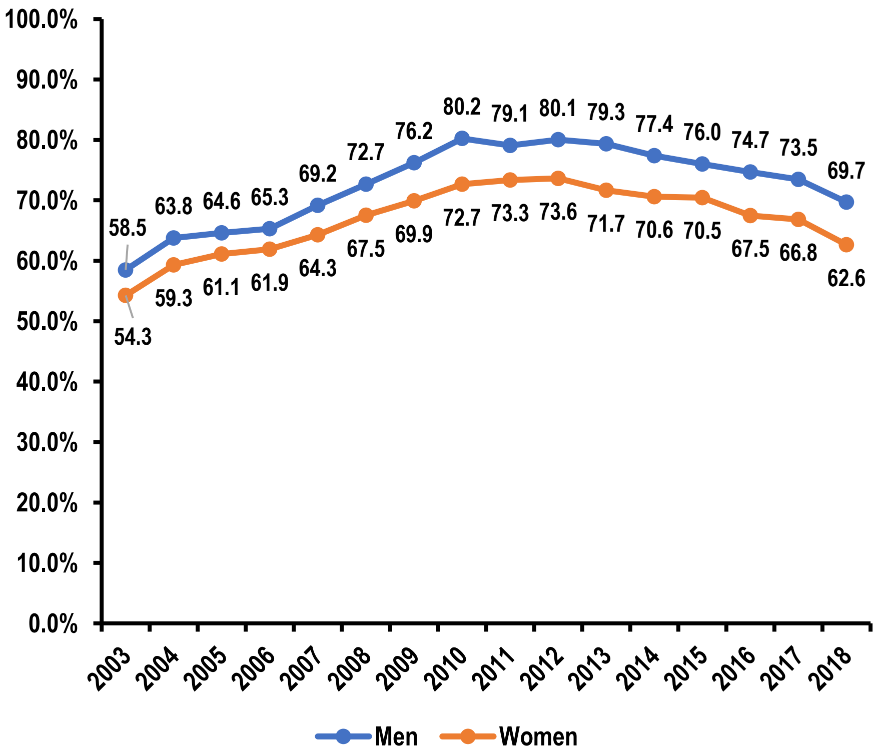

Supplementary Figure 4. Prescription Rate of Statins at Discharge in Patients with AMI undergoing PCI

A. Total patients with AMI

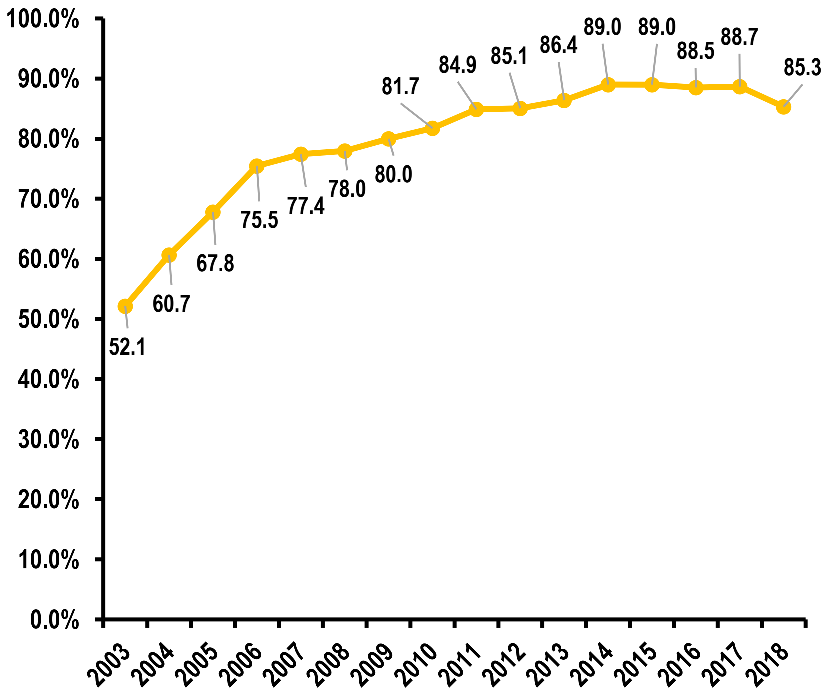

B. Gender difference

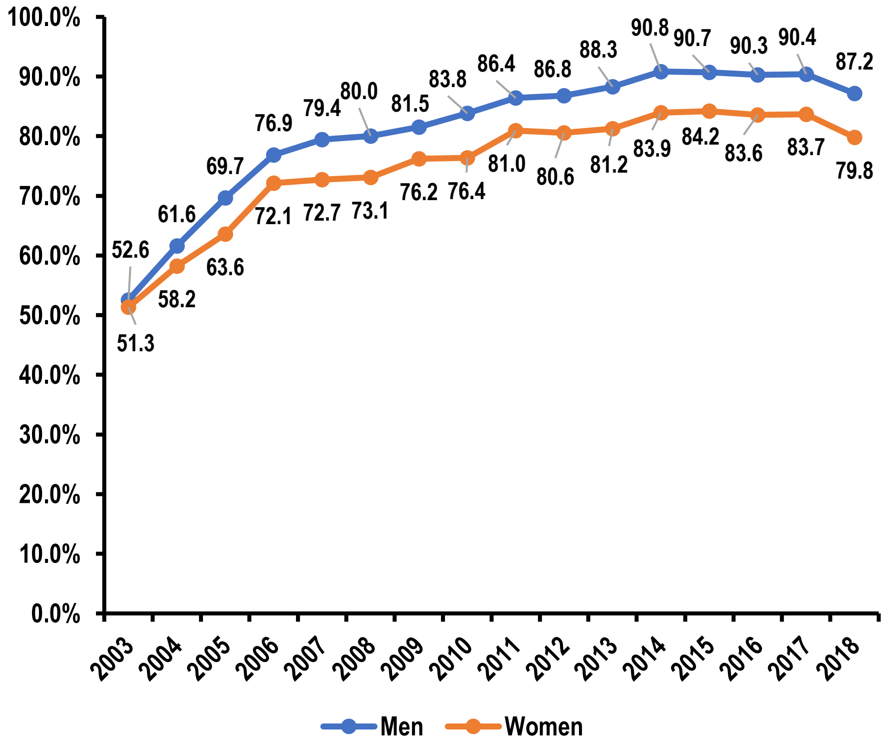

Supplementary Figure 5. Clinical Outcomes of Patients with AMI from 2003 to 2018 according to the Korean National Health Insurance Claims Database

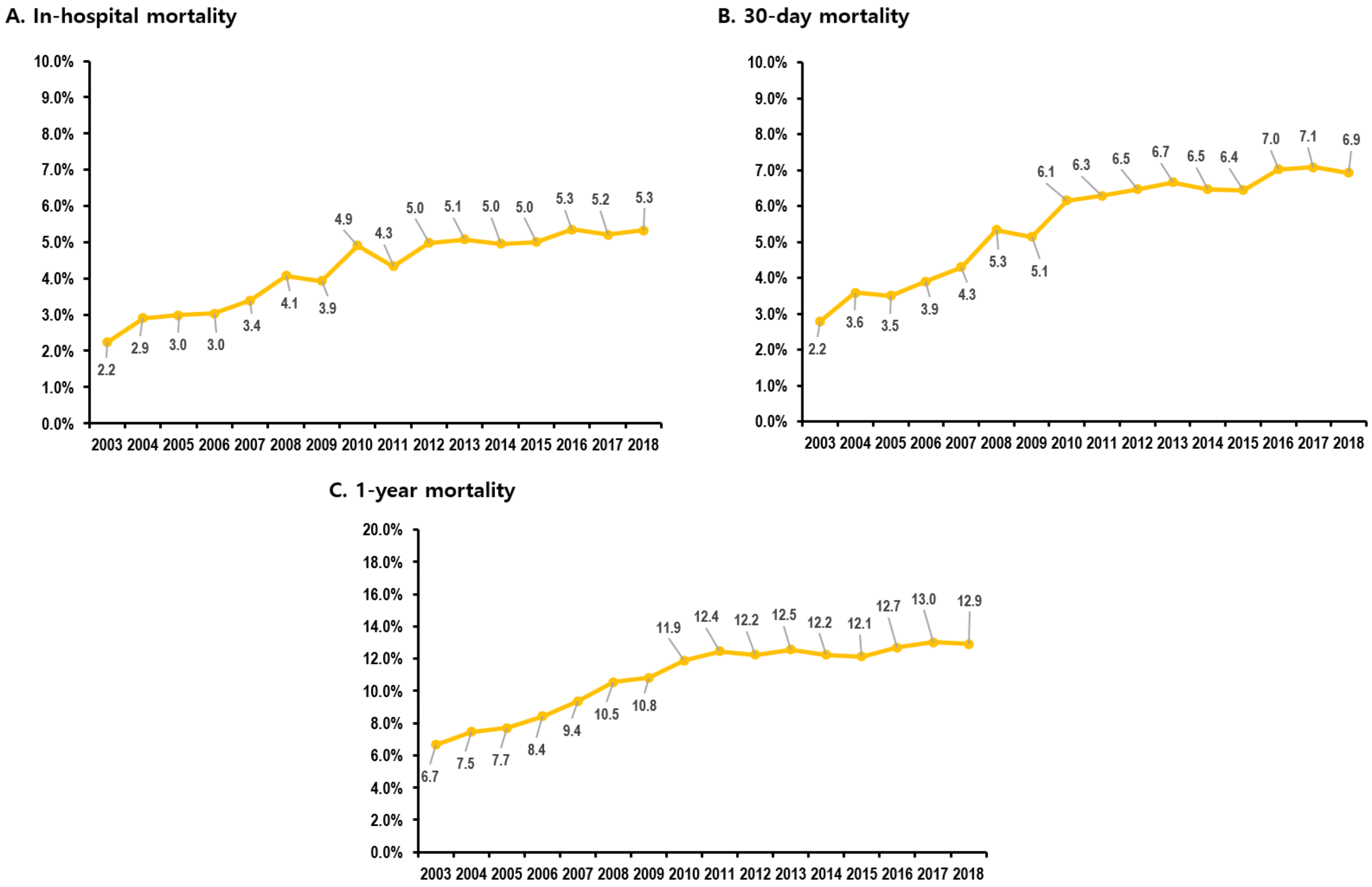

In-hospital, 30-day, and 1-year mortality rates have increased since 2011, reaching 5.3%, 6.9%, and 12.9% in 2018, respectively.
